# Swallow contrast-enhanced ultrasonography detects a tiny cervical esophageal diverticulum missed on barium esophagography: a case report

**DOI:** 10.3389/fmed.2026.1843349

**Published:** 2026-08-26

**Authors:** Feng Yu, Yaoyao Li, Shengting Chen, Chengxiang Zhou

**Affiliations:** Department of Ultrasound, Clinical Medical College and the First Affiliated Hospital of Chengdu Medical College, Chengdu, China

**Keywords:** barium esophagogram, esophageal diverticulum, SCEUS, swallow contrast-enhanced ultrasonography, thyroid nodules

## Abstract

**Background:**

Barium esophagography is standard for esophageal lesions but may miss subtle small diverticula. Prior literatures have reported swallow contrast-enhanced ultrasonography (SCEUS) for esophageal diverticulum detection. This case presents a tiny cervical esophageal diverticulum misclassified as C-TIRADS 4A thyroid nodule, undetected by barium esophagography but visualized by SCEUS. We describe the imaging characteristics and incremental value of microbubble contrast for this easily misdiagnosed entity.

**Case presentation:**

We report the case of a 38-year-old Chinese man with no initial clinical symptoms related to the esophagus. In June 2019, a routine ultrasonographic examination revealed a solid nodule easuring approximately 5. 1 × 3.5 mm in the posteromedial aspect of the middle and upper pole of the left thyroid lobe. This nodule demonstrated close anatomical proximity to theadjacent esophageal wall, raising suspicion for an esophageal origin. Subsequent barium esophagography was performed, which demonstrated no significant abnormalities. Given the absence of related clinical symptoms, the patient was placed on an annual follow-up protocol. On January 18, 2025, conventional gray-scale ultrasonography revealed a 1-mm increase in the maximum diameter of the lesion compared with the initial examination, prompting further evaluation with SCEUS, which clearly confirmed the presence of an esophageal diverticulum in the upper-middle third of the left thyroid lobe measuring 5.6 × 3.8 mm in size. For intuitive visual comparison, one healthy volunteer was enrolled to acquire standard SCEUS images depicting normal physiological swallowing morphology.

**Conclusion:**

In this single case, SCEUS visualized a tiny cervical esophageal diverticulum undetected by barium esophagography, helping avoid unnecessary thyroid resection. Microbubble contrast provides dynamic real-time delineation of diverticular communication that cannot be fully resolved by plain water swallow ultrasound. However, this single observation cannot support generalized superior diagnostic performance of SCEUS. Further cohort studies are needed to validate the clinical utility of SCEUS for small esophageal diverticula mimicking thyroid nodules.

## Background

Esophageal diverticulum is a rare disease of the esophagus with an estimated incidence of <1% in thegeneral population (1). The condition is classified according to its location in the esophagus as proximal (Zenker’s or pharyngoesophageal, 70% of all cases), mid-esophageal (thoracic or mediastinal, 10% of all cases), or distal (epiphrenic, 20% of all cases) (2). The standard diagnostic approaches for esophageal diverticula primarily include barium esophagography and endoscopic evaluation (3). Barium esophagography has a diagnostic sensitivity of 95%, with reported false-negative rates of up to 5% (4). With the increasing clinical utilization of contrast-enhanced ultrasonography (CEUS) across various medical specialties, its diagnostic value for esophageal diverticula has been increasingly recognized. Compared with conventional barium esophagography, SCEUS offers distinct advantages over barium esophagography, including no radiation, real-time dynamic evaluation, and bedside availability (5). In this case report, no significant abnormalities were noted on barium esophagography, whereas SCEUS successfully diagnosed an esophageal diverticulum. This imaging modality could serve as an auxiliary evaluation method for esophageal lesionsand lesions involving adjacent structures.

## Case presentation

**Figure 1:**
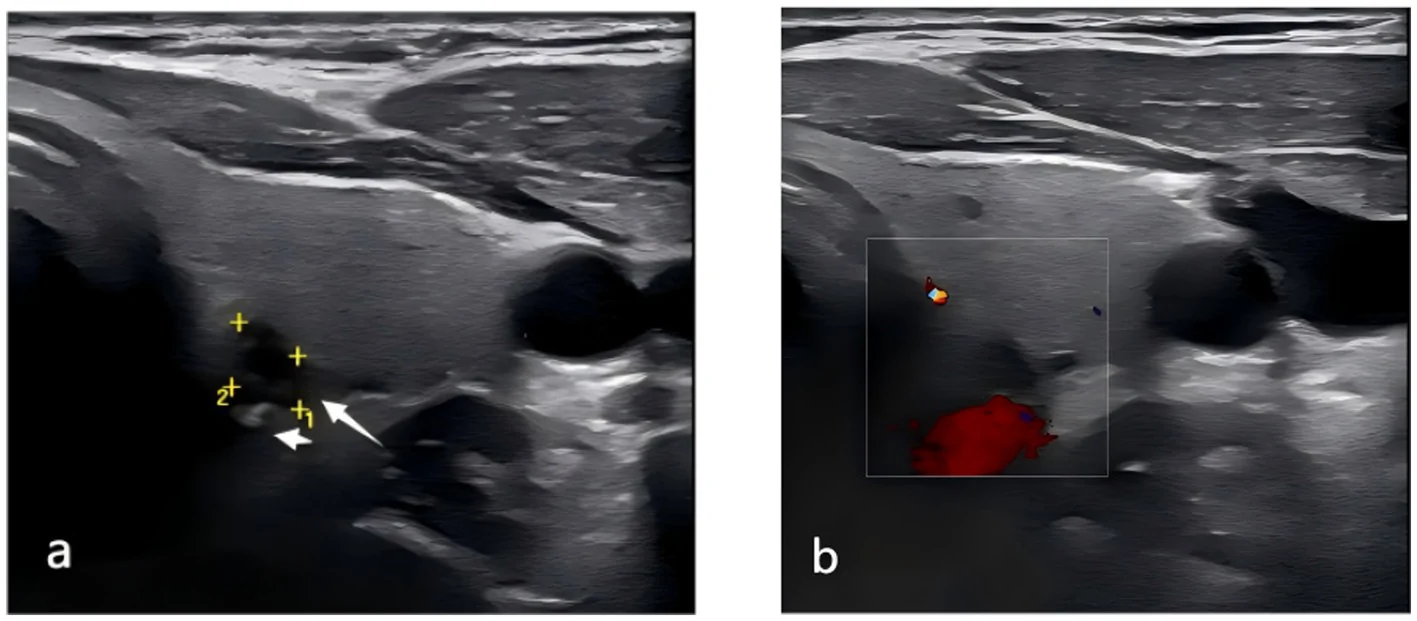
Thyroid gray-scale ultrasound examination and color Doppler flow imaging of a 38-year-old male patient. **(a)** The gray-scale ultrasound examination revealed a hypoechoic nodule located in the posterior-medial part of the left lobe of the thyroid gland (indicated by the arrow), with a size of 5.6 × 3.8 mm. The edge of the nodule was clear, and there were punctate hyperechoic areas (indicated by the short arrow) in the posterior-medial part. **(b)** The color Doppler imaging showed no vascular distribution within the nodule.

**Figure 2:**
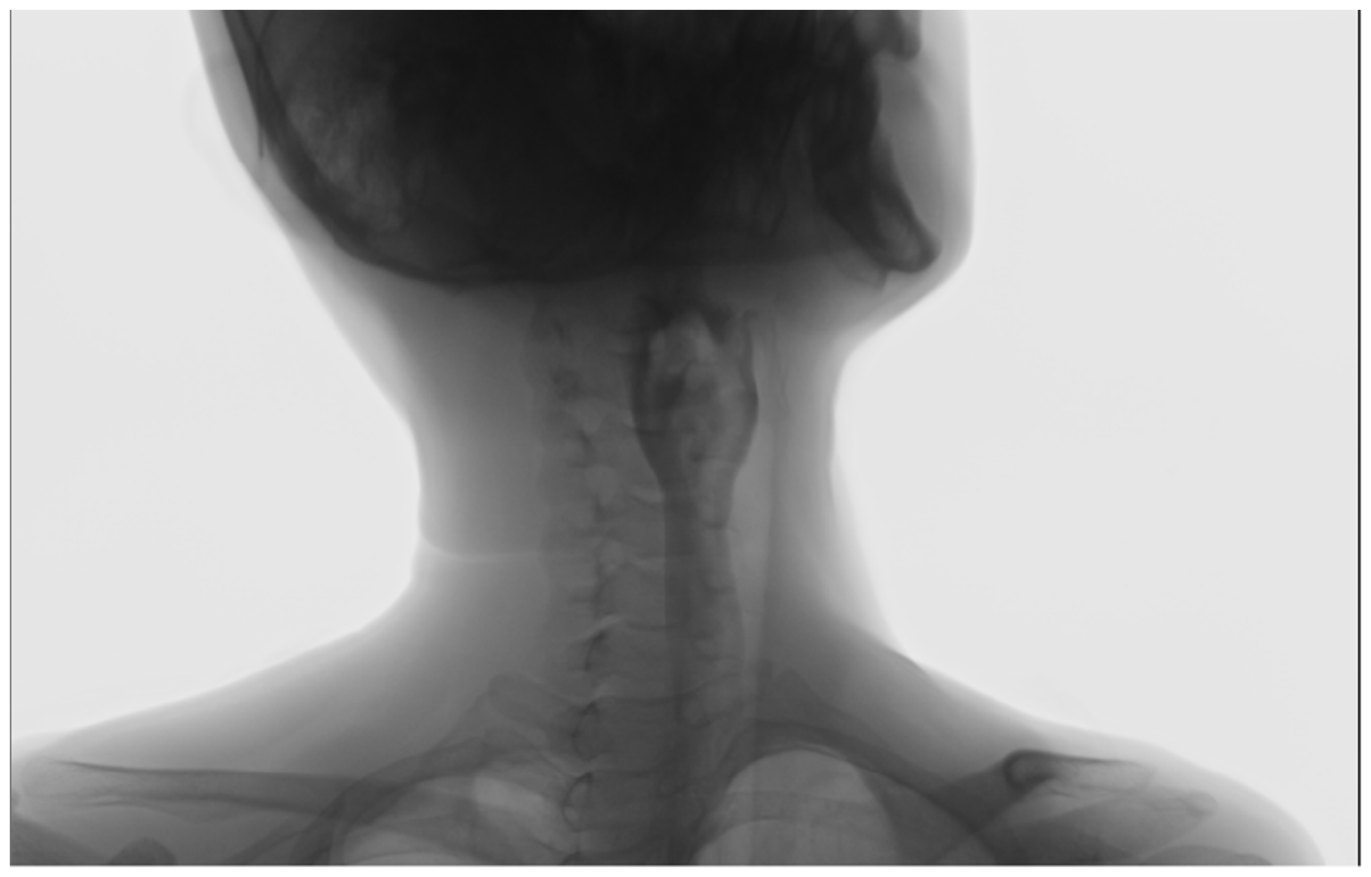
Barium swallow study demonstrates an unremarkable esophagus.

**Figure 3:**
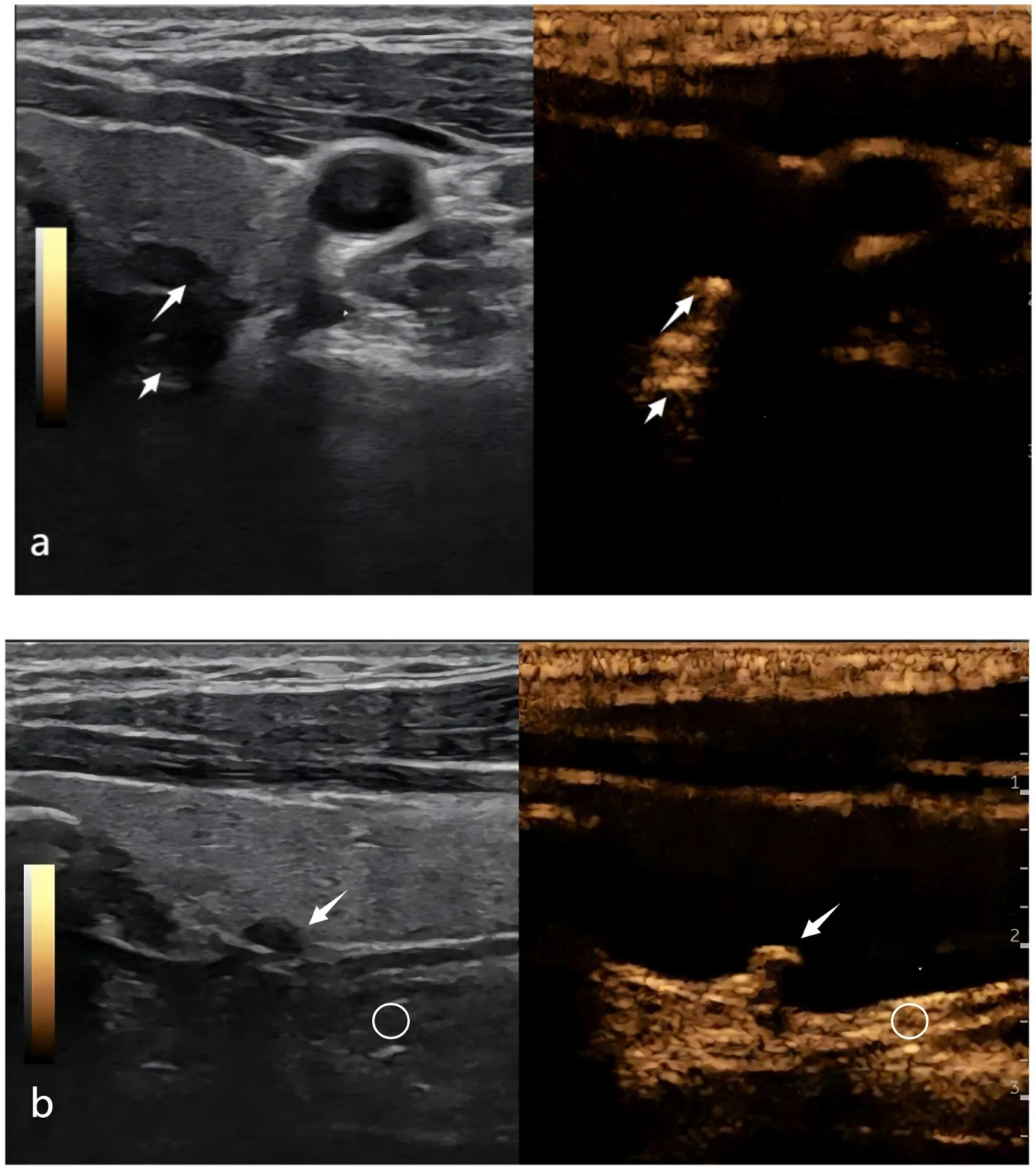
Still image from Supplementary Video S1 (Patient). **(a)** Transverse view: Upon contrast ingestion, concurrent microbubble filling is observed in the hypoechoic lesion and esophageal lumen (arrow), while the thyroid parenchyma remains unenhanced. Left: Gray-scale correlate. **(b)** Longitudinal view: This section delineates a continuous enhanced pathway (arrows) linking the lesion to the esophagus (circle), with both structures showing uniform hyperenhancement, confirming patency. Left: Gray-scale correlate. Arrow: lesion; Circle: normal esophagus.

**Figure 4:**
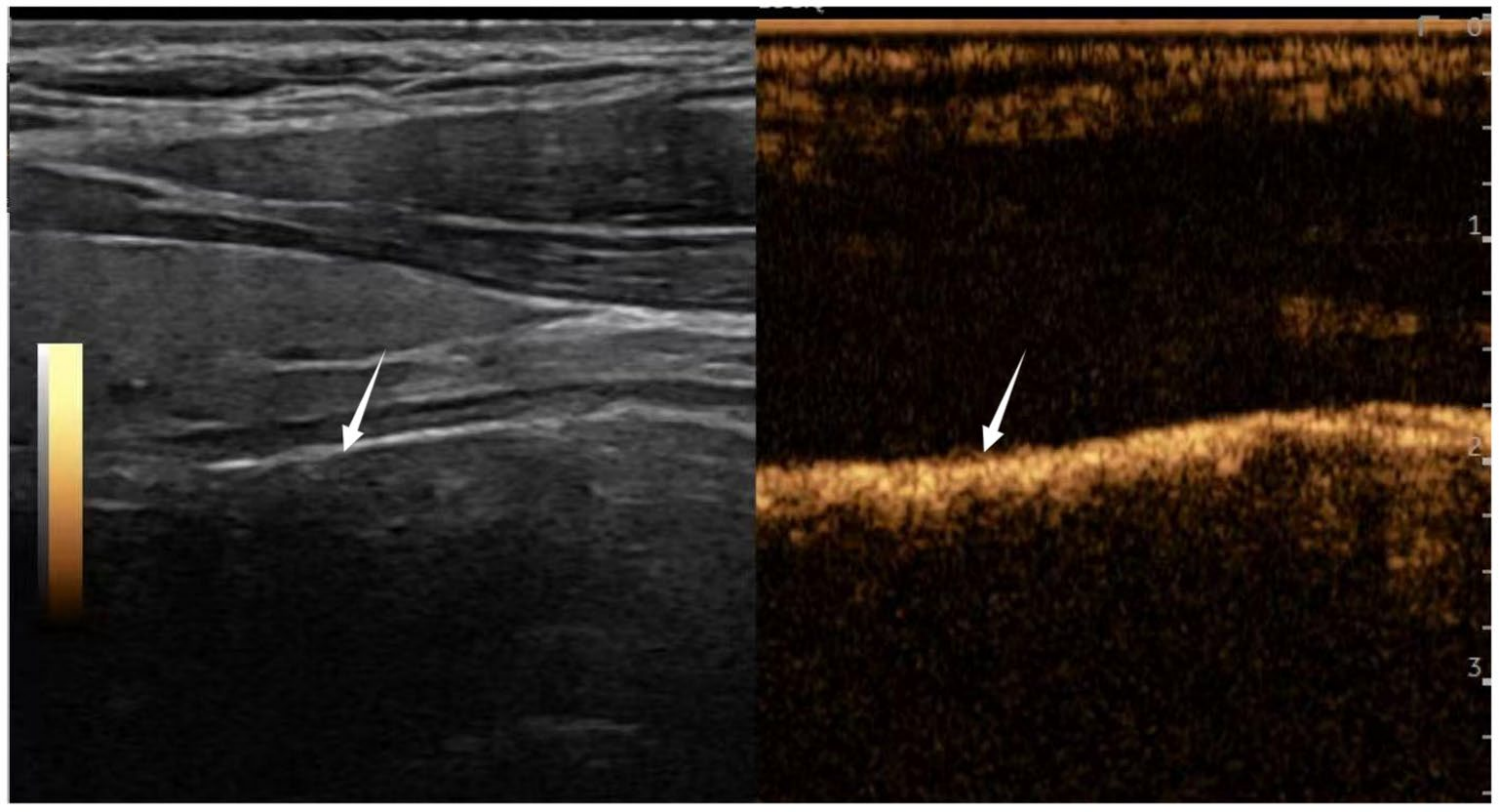
Still image from Supplementary Video S2 (Normal control). The well-defined borders of the contrast agent indicate a continuous and intact esophageal wall structure, with no evidence of focal outpouching.

**Figure 5:**
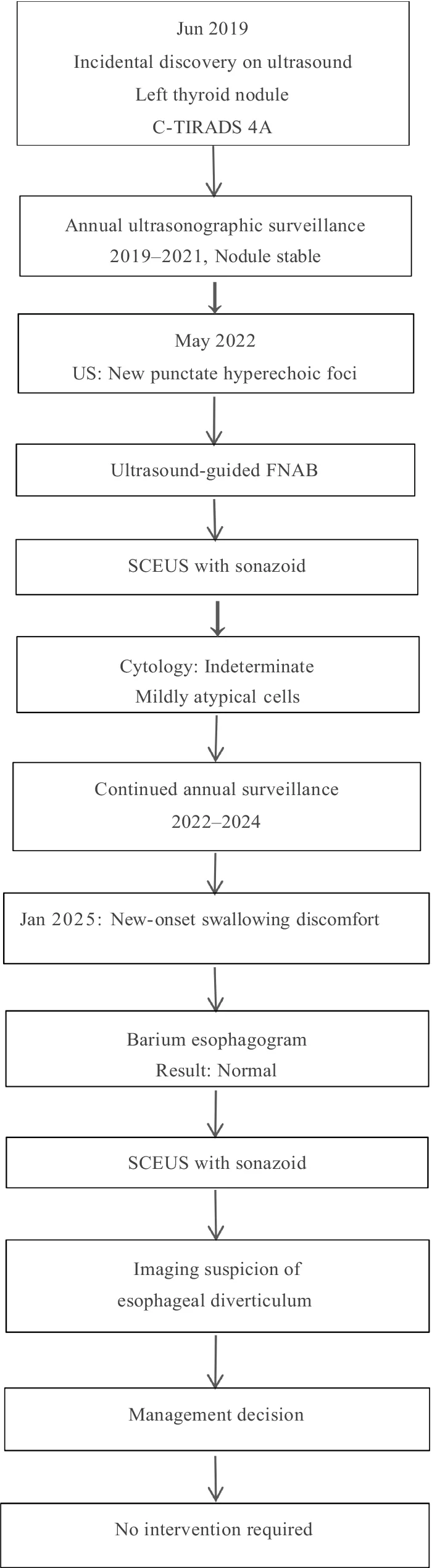
Clinical flowchart demonstrating the patient’s clinical course: incidental detection of the suspected thyroid nodule in 2019, regular surveillance, fine‑needle aspiration cytology, barium esophagography, swallow contrast‑enhanced ultrasonography diagnosis of cervical esophageal diverticulum, and final decision for periodic follow‑up without surgical intervention in 2025.

In January 2025, a 38-year-old Chinese man presented to our hospital with a 6-year history ofthyroid nodules and recent-onset discomfort upon swallowing. The patient’s family, genetic and psychosocial histories were all negative, and he had no history of relevant upper gastrointestinal interventions or adverse sequelae. Complete physical examinations covering general status, neck, gastrointestinal tract, cardiopulmonary, musculoskeletal and neurological systems yielded entirely unremarkable findings with no suspicious positive signs. The nodule had initially been identified during routine thyroid ultrasonography (using an ML6-15 high-frequency linear transducer on a GE LOGIQ E20 color Doppler ultrasound system, GE Medical Systems, Milwaukee, WI, USA) in June 2019 and was characterized as a 5.6 × 3.8 mm solid lesion located in the upper-middle third ofthe left thyroid lobe. The well-circumscribed nodule exhibited regular margins, a longitudinal-to-transverse diameter ratio of >1, and uniform hypoechogenicity. Accordingly, it was categorized as a level 4A under the Chinese thyroid imaging reporting and data system (C-TIRADS), indicating a suspicion of malignancy. No specific intervention was administered at that time. The patient remained asymptomatic during follow-up but elected to undergo annual ultrasonographic surveillance because of persistent concerns regarding the nodule’s characteristics. In the follow-up examination in May 2022, thyroid ultrasonography revealed punctate hyperechoic foci within the nodule (Figure 1). For diagnostic clarification, the patient underwent ultrasonography-guided fine-needle aspiration biopsy (FNAB), and cytopathological examination revealed mildly atypical follicular epithelial cells, prompting concern. In January 2025, the patient occasionally experienced discomfort upon swallowing and requested a barium esophagogram to rule out the possibility of esophageal lesions associated with the thyroid nodule. The patient subsequently underwent a swallowing examination with a type II barium sulfate suspension (1,3 barium-to-water ratio) with imaging performed on the X-ray system (PLD9600; Perlove, Zhuhai, China). The results were normal (Figure 2). Considering the prolonged duration of the nodule and documented cytological atypia, a comprehensive evaluation of the nodule characteristics and anatomical relationships seemed prudent. After a detailed physician–patient discussion, the patient consented to enhanced ultrasonography with Sonazoid (0.00015 mL/kg, GE Healthcare, Oslo, Norway) to obtain definitive diagnostic information. Following a 6-h fast and after providing written informed consent, the patient underwent the examination. Sonazoid powder was reconstituted with 2 mL of sterile water for injection, 1 mL of the resulting suspension was mixed with 100 mL of room-temperature purified water in a 250-mL cup and gently agitated for 1 min to ensure homogeneity. The patient was placed in the supine position with neck hyperextension for ultrasonographic examination. Thereafter, he retained 5 mL of contrast suspension orally. Gray-scale imaging was performed in the preset thyroid mode using an ML6-15 high-frequency linear transducer for real-time scanning. Upon identifying the lesion at the mid-upper pole of the left thyroid lobe, contrast-enhanced mode was initiated with the mechanical index (MI) set at 0.24, which preserves microbubble integrity during real-time evaluation. The patient was instructed to swallow the contrast agent at a controlled rate of approximately 5 mL/s while the sonographer observed the contrast enhancement patterns ofthe lesion and acquired dynamic images. Real-time CEUS revealed dynamic inflow ofthe contrast agent into the lesion via a narrow channel from the esophageal lumen, with rapid, intense enhancement ofthe channel while the surrounding hypoechoic area remained non-perfused (see Supplementary Video S1, patient). A representative still image captured at peak enhancement is shown in Figure 3. The lesion was demarcated from the adjacent thyroid gland, with no contrast uptake in the thyroid gland. These imaging findings enabled lesion characterization, though a definitive diagnosis could not be confirmed without additional gold-standard examinations. To provide visual reference for normal swallowing appearances, one age-matched healthy volunteer was enrolled in this study. The normal swallowing process is presented in Video S2, with a corresponding still image in Figure 4. After a 1-week follow-up period, complete resolution of the swallowing discomfort was noted. This transient symptom was attributed to temporary pharyngeal inflammation rather than diverticulum. Given the small size ofthe diverticulum, no further intervention was needed. Instead, the patient was placed on a follow-up protocol involving SCEUS every 2 years to monitor for changes in diverticulum size (see Figure 5).

## Discussion and conclusions

Cases of esophageal diverticulum are characterized by localized outpouching ofthe esophageal wall and are very rarely observed. Patients with an esophageal diverticulum present with a sac-like structure that maintains communication with the esophageal lumen. The pathogenesis ofthis condition involves impaired esophageal muscle relaxation and motility dysfunction, leading to elevated intraluminal pressure, with potential subsequent herniation ofthe mucosa and submucosa through anatomically weakened areas. The clinical presentation of such cases varies according to the size and location ofthe diverticula. Most esophageal diverticula remain small and clinically asymptomatic, being incidentally detected during other routine diagnostic procedures. However, when symptoms do occur, they are most pronounced in cases of distal diverticula (4). The predominantclinical manifestation include dysphagia with gastroesophageal reflux; moreover, as the diverticulum grows, there is progressive compression of adjacent anatomical structures (5). This condition isprimarily diagnosed through radiographic evaluation.

Barium esophagography is generally regarded as the diagnostic gold standard for esophageal diverticula owing to its ability to enable the dynamic visualization of diverticular morphology and precise location through barium contrast flow patterns. However, this modality has several inherent limitations. First, as a fluoroscopic examination, it entails exposure to ionizing radiation, which is of particular concern in pediatric and obstetric patients. Second, spatial resolution is inherently limited by conventional fluoroscopy technology. Third, inadequate mucosal coating or rapid esophageal transit may result in failure to detect subtle pathologies such as small diverticula, leading to diagnostic inaccuracies. Oral CEUS enables the detection ofminute diverticula that might escape detection on conventional esophagography (6). Other diagnostic modalities including phagogastroduodenoscopy (EGD), cervical/thoracic CT, and esophageal manometry are available for identifying esophageal diverticula, each with distinct advantages and limitations relative to SCEUS. Endoscopy can directly visualize mucosal diverticular lining and exclude malignant lesions, yet it fails to dynamically display the real-time swallowing communication between the esophageal lumen and small outpouchings, and may miss tiny cervical diverticula due to limited field of view. CT enables full anatomical assessment of thoracic esophageal segments obscured by ultrasound acoustic artifacts but involves ionizing radiation and cannot capture dynamic contrast filling during deglutition. Esophageal manometry evaluate esophageal motility dysfunction underlying diverticulum formation; however, it provides no morphological imaging of the diverticular sac itself. In the present case, barium esophagography revealedno significant abnormalities. However, the patient’s clinical presentation led to the suspicion that anatomicalvariations, such as rapid diverticular emptying and small size, led to a false-negative diagnosis. Pathologic analysis identified scattered thyroid follicular cells, consistent with incidental translocation during FNA (7). During the dynamic CEUS evaluation ofswallowing, divergences from normal esophageal anatomy characteristic of diverticula were observed. A single age-matched healthy volunteer was enrolled to demonstrate standard SCEUS findings during physiological swallowing. The volunteer was male. Physical examination and basic physiological parameters were within normal ranges. Recruitment was conducted through publicly advertised announcements at our institution. All procedures were in compliance with ethical standards and were performed with the volunteer’s informed consent. Imagingobtained via SCEUS in the volunteer revealed tubular esophageal structures with uninterrupted wall continuity, smooth mucosal lining, with no evidence of focal outpouching (Figure 4). Following deglutition, the contrast microbubbles rapidly traversed the lumen, initially demonstrating homogeneous high enhancement. Under the combined effects of esophageal peristalsis and the gravity ofthe contrast agent suspension, the contrast agent gradually cleared within a 120-s period. As reported in a prior studyl (8), microbubble fragmentation is primarily induced by luminal pressure changes and mucosal contact. This phenomenon results in a gradual decline in echo intensity, which aligns with physiological clearance patterns. In contrast, within small diverticula measuring less than 1 cm in diameter, contrast microbubbles tend to accumulate in the lateral wall ofthe outpouching, potentially forming a persistent hyperechoic focus during the contrast-enhanced examination (9). Three pathophysiological factors contributed to this phenomenon: a narrow diverticular neck,a lack of effective peristalsis and diminished intracavitary hydrostatic pressure. Together, these prolonged microbubble retention times (10). However, conventional water-swallow sonography merely produces transient faint echogenic contrast with rapid microbubble washout, making it unable to clearly outline the narrow necks of tiny esophageal diverticula or identify contrast retention within diverticular pouches measuring less than 1 cm in diameter. For comparison, SCEUS images acquired from a single healthy volunteer were used as visual references for normal esophageal swallowing physiology. By visually comparing the SCEUS images of normal swallowing obtained from this healthy volunteer with those of the patient, two imaging manifestations of esophageal diverticulum in this case were identified: static morphological changes including focal contrast hyperenhancement and localized outward bulging of the esophageal wall, as well as dynamic imaging features characterized by delayed microbubble clearance. Notably, small esophageal microdiverticula are frequently misidentified as thyroid nodules on routine thyroid ultrasound. This diagnostic pitfall arises due to anatomical positioning: the esophagus runs posteromedial to the thyroid gland within the tracheoesophageal groove, and its wall intrinsically presents a hypoechoic appearance under ultrasound. Additionally, gas within the esophageal diverticulum can present as punctate hyperechogenicity on ultrasonography, a sonographic appearance that can mimic calcific foci (11). Failure to recognize the characteristic location and sonographic features of an esophageal diverticulum may lead to its misdiagnosis as a malignant thyroid nodule, potentially leading to unnecessary surgical intervention. The misdiagnosis of an esophageal diverticulum as malignant thyroid nodules can place a considerable psychological burden on the patient, and inappropriate decision- making regarding resection or ablation is likely to cause esophageal damage. Consequently, accuratelydetermining the lesion’s origin and detecting small, atypical esophageal diverticula pose significant clinical challenges. Compared with conventional barium esophagography, ultrasonography offers several distinct advantages, including the absence of the need for exposure to ionizing radiation, excellent reproducibility of results, real-time dynamic imaging, and simultaneous visualization of thyroid morphology. Furthermore, SCEUS allows for detailed observation of the real-time dynamics of intraluminal contrast flow, alongside the specific patterns of contrast enhancement and washout within esophageal diverticula. The application of SCEUS significantly improved the detection sensitivity and diagnostic accuracy for small esophageal diverticula, thereby preventing misdiagnosis as thyroid nodules and subsequent unnecessary interventions such as ablation or surgery. This imaging modality holds considerable promise for broader application in diagnosing other esophageal conditions. Following contrast agent administration, we found that dynamic ultrasonography revealed three characteristic findings: (1) contrast transit from the esophageal lumen to the diverticulum dumen, (2) progressive contrast accumulation, and (3) clearance was observed only after a retention time of more than 30 s. These pathognomonic features were used to confirm our definitive diagnosis of esophageal diverticulum. SCEUS comprehensively evaluates the anatomy ofthe diverticulum and its surroundings and can help exclude malignancy by defining the interface between the contrast and the wall. In this case, the unperfused hypoechoic rim around the diverticulum was consistent with the esophageal wall. The clear and smooth border of the contrast column suggested a benign, smooth-walled diverticulum. Therefore, integrating high-frequency ultrasound with CEUS enhances diagnostic accuracy for esophageal diverticula and minimizes diagnostic errors.

Based on their anatomical location, esophageal diverticula can be classified as pharyngeal (Zenker), mid-esophageal, or epiphrenic diverticula (12). Pharyngeal diverticula are the most prevalent, and theytypically occur at the junction ofthe cricopharyngeal (CP) and inferior pharyngeal constrictor muscles. This type of mucosal herniation, which spares the muscular layer, is considered as a pseudodiverticulum. Similar mucosal protrusions can develop at other esophageal sites, including the Killian—Jamieson area between the oblique and transverse CP muscle fibers, and Laimer’s triangle between the CP muscle and esophageal musculature (13–15). Diverticula in the Killian-Jamieson area appear as anterolateral cervical diverticula. These are frequently mistaken for thyroid nodules onultrasonography, with intraluminal gas bubbles misinterpreted as the microcalcifications characteristic of thyroid malignancies, particularly papillary thyroid carcinoma. The similarities of these presentations underscore the importance of SCEUS for accurate differentiation (16, 17). SCEUS is a reliable diagnostic method for pharyngoesophageal diverticula (10). Mid-esophageal diverticula are predominantly pseudodiverticula, typically secondary to esophageal inflammation or resulting from congenital malformations (18). The wide diverticular orifice of pharyngoesophageal diverticula and rapid contrast agent washout can limit its detection on sonography. However, high-frequency ultrasonography with SCEUS enables the comprehensive visualization of the diverticular morphology and real-time dynamic assessment, significantly improving diagnostic accuracy. Ultrasound contrast agents have evolved to the second generation, mainly represented by SonoVue and Sonazoid. Although SonoVue is commonly adopted for oral contrast-enhanced ultrasonography in previous researches, Sonazoid was used in the present study due to its more stable microbubbles and longer enhancement duration, which are suitable for high-frequency (≥15 MHz) cervical ultrasonography (19, 20). Microbubble contrast agents are mostly administered intravenously, with limited extravascular applications such as vesicoureteral reflux (21) and hysterosalpingography imaging (22). Sonazoid is supplied as lyophilized powder for injection with non-toxic perfluorobutane microspheres. Compared with iodinated contrast media, it exhibits a better safety profile with rare severe allergic reactions. Clinical trials at home and abroad have validated its mild adverse reactions and favorable tolerability, supporting its safe use in oral swallow contrast-enhanced ultrasonography (23–27).

The combination of SCEUS and high-frequency ultrasonography demonstrates significant diagnostic value for pharyngoesophageal diverticula. It enables a clear visualization of both the diverticular morphology and its anatomical connection to the esophageal lumen. However, its diagnostic accuracy for mid-esophageal and supradiaphragmatic diverticula may be influenced by factors such as the dimensions and anatomical location ofthe diverticulum, as well as interpatient variability. The esophagus originates at the inferior margin of the pharynx, corresponding to the level ofthe sixth cervical vertebra (C6). It extends inferiorly from C6 to the level ofthe eleventh thoracic vertebra (T11) and is anatomically divided into cervical, thoracic, and abdominal segments along its course (28). The thoracic segment ofthe esophagus within the mediastinum cannot be adequately assessed by ultrasonography due to acoustic interference from the rib cage and lung tissue. Therefore, the use of this modality is limited to visualizing the cervical and abdominal esophageal segments (29). Consequently, SCEUS does not demonstrate equivalent diagnostic efficacy for all classifications of esophageal diverticula. When indicated, complementary imaging modalities such as barium esophagography or cross-sectional imaging should be used for diagnostic precision and reliability. A limitation of this report is the absence of independent confirmatory testing via endoscopy, other imaging modalities such as CT, and repeated targeted fluoroscopy; the diverticulum was diagnosed exclusively based on characteristic dynamic SCEUS imaging findings. Esophageal diverticula are rare and benign. Our case demonstrates that SCEUS has superior diagnostic performance to conventional barium esophagography in the detection of small esophageal diverticula. This technique not only facilitates the correct identification of cases previously misdiagnosed as thyroid nodules on conventional ultrasonography but also markedly reduces the incidence of missed diagnosis of esophageal diverticula. Our findings also validate the distinct advantages of orally administered ultrasonography contrast agents in the diagnostic evaluation of esophageal pathologies.

## Data Availability

The original contributions presented in the study are included in the article/Supplementary material, further inquiries can be directed to the corresponding author.
